# Low-dose daratumumab and bortezomib for the treatment of proliferative glomerulonephritis with monoclonal immunoglobulin deposits: a case report

**DOI:** 10.3389/fmed.2026.1899020

**Published:** 2026-08-14

**Authors:** Jiaomei Zhu, Limin Zhang, Huibo Zhao, Li Tian, Huaying Pei, Shaomei Li, Lingling Xing

**Affiliations:** Department of Nephrology, The Second Hospital of Hebei Medical University, Shijiazhuang, China

**Keywords:** CyBorD-refractory, daratumumab, dose de-escalation, monoclonal gammopathy of renal significance, proliferative glomerulonephritis with monoclonal immunoglobulin deposits

## Abstract

**Background:**

Proliferative glomerulonephritis with monoclonal immunoglobulin deposits (PGNMID) is an exceptionally rare glomerular disease, accounting for only 0.17–3.7% of all renal biopsies, and no consensus–based treatment algo-rithm has been established. Although daratumumab, a humanized anti-CD38 monoclonal antibody, has shown promising efficacy in PGNMID, the existing evidence is limited to small case series using standard-dose regimens (16 mg/kg). Critically, data on reduced-dose daratumumab in elderly patients with CyBorD-refractory disease and multiple comorbidities remain scarce. This case report adds to the limited experience with a reduced-dose strategy in a high-risk elderly patient with CyBorD-refractory PGNMID.

**Case presentation:**

A 73-year-old Chinese male with biopsy-proven PGNMID (IgG3-*κ* subtype), chronic kidney disease stage 3a, a baseline serum creatinine 1.5 mg/dL (132.6 μmol/L), proteinuria 5.61 g/24 h, and multiple comorbidities (hypertension, coronary artery disease, and prior cerebral infarction) received first-line CyBorD therapy (cyclophosphamide, bortezomib, and dexamethasone). After four cycles, he demonstrated treatment failure. He subsequently developed progressive renal deterioration [serum creatinine 2.7 mg/dL (239 μmol/L)], severe nephrotic-range proteinuria (10.61 g/24 h), hypoalbuminemia (22.8 g/L), and decompensated heart failure. Given his advanced age and high infection risk, a risk-adapted regimen was administered monthly for 12 cycles: reduced-dose daratumumab (400 mg, ∼6 mg/kg, 37.5% of the standard 16 mg/kg dose) combined with bortezomib 2.2 mg. After 12 months, the patient achieved partial renal remission: proteinuria decreased to 1.12 g/24 h, serum albumin normalized to 38.1 g/L, serum creatinine stabilized at 1.6 mg/dL (141.4 μmol/L), immunofixation electrophoresis converted to negative, and clinical resolution of heart failure was achieved. Notably, no infectious complications were documented throughout the treatment period.

**Conclusion:**

This case suggests that low-dose daratumumab combined with bortezomib may be a feasible and apparently well-tolerated salvage option in this selected patient with CyBorD-refractory PGNMID and a heavy comorbidity burden. The favourable outcome in this single case raises the possibility that dose de-escalation might preserve efficacy while mitigating toxicity, although this hypothesis requires confirmation in larger studies. This report adds to the limited global evidence for daratumumab in PGNMID and highlights the need for personalized, risk-stratified dosing strategies in this rare and challenging glomerulopathy.

## Introduction

Proliferative Glomerulonephritis with Monoclonal Immunoglobulin Deposits (PGNMID), formally defined in 2004, is recognized as a distinct subtype of Monoclonal Gammopathy of Renal Significance (MGRS) and has long posed a formidable challenge in clinical nephrology (1–3). It is characterized by the selective deposition of monoclonal immunoglobulins in the glomeruli, without systemic involvement. The condition predominantly affects middle-aged adults and typically presents with proteinuria, haematuria, and renal insufficiency (1–4). The IgG3-*κ* subtype is the most common immunophenotype, accounting for 60–87% of cases (1, 5).

Clonally directed therapy is the cornerstone of PGNMID management. For plasma cell clones, bortezomib-based regimens such as CyBorD (cyclophosphamide, bortezomib, dexamethasone) are recommended as first-line therapy, while rituximab-based protocols target B-cell clones (6, 7). Nevertheless, treatment resistance remains a significant challenge (8), particularly in elderly patients with comorbidities who face elevated risks of adverse events and disease progression. Therapeutic options for such refractory cases remain limited, highlighting an urgent unmet clinical need for alternative treatment agents.

Daratumumab, a humanized IgG1κ monoclonal antibody targeting CD38, has emerged as a transformative therapy in multiple myeloma (MM) and systemic light chain amyloidosis (9, 10). Beyond direct cytolytic effects, recent evidence suggests that daratumumab modulates the renal immune microenvironment by eliminating immunosuppressive cells, creating a favorable milieu for renal repair (9). Preliminary data from small–sample studies and case reports have demonstrated promising efficacy in PGNMID (11, 12), but data on reduced-dose regimens in elderly, high-risk patients with CyBorD-refractory disease remain scarce. Recently, a landmark study by Javaugue et al. (13) using high-throughput immunoglobulin repertoire sequencing challenged the traditional view, suggesting that PGNMID-IgG3*κ* may not always represent a typical clonally driven MGRS entity but rather an immune-mediated oligoclonal glomerulopathy. While this novel hypothesis provides important insights into the pathogenesis of a subset of PGNMID, it remains a subject of ongoing debate and has not yet been incorporated into international consensus guidelines. Regardless of this debate, plasma cells or plasmablasts remain the source of pathogenic immunoglobulins, making CD38 targeting with daratumumab—even at reduced doses—a rational therapeutic strategy. This case report describes our experience with low-dose daratumumab in an elderly, high-risk patient with PGNMID who intolerant of the CyBorD regimen, adding to the limited evidence on dose de-escalation in this challenging population.

Herein, we present a case of a 73–year–old male with PGNMID (IgG3–*κ* subtype) and CKD Stage 3a who failed to respond to first-line CyBorD therapy (bortezomib + cyclophosphamide + dexamethasone) and subsequently developed progressive renal dysfunction, severe edema, and heart failure. He was treated with low–dose daratumumab (400 mg, ∼ 6 mg/kg) combined with bortezomib, resulting in significant reductions in proteinuria, stabilization of renal function, and clinical resolution of heart failure. This case adds to the limited anecdotal evidence for daratumumab in refractory PGNMID and highlights the need for further investigation of dose deescalation strategies in high-risk populations. By documenting this single-case observation, we aim to generate hypotheses that may inform future clinical investigation of personalized dosing strategies in high-risk populations.

## Case presentation

### Baseline information and laboratory tests

A 73–year–old Chinese male presented to the Second Hospital of Hebei Medical University with facial and lower limb edema persisting for 2 years. The patient weighed 69 kg and measured 160 cm in height.

Laboratory tests revealed: hematuria 2+, proteinuria 4+, serum albumin 36.6 g/L, serum creatinine 1.5 mg/dL (132.6 μmol/L), and hemoglobin 97 g/L. Twenty–four–hour urine protein quantification was 5.61 g/24 h. Serum free light chain analysis showed: *κ* 42.10 mg/L, and *λ* 46.80 mg/L, with a *κ*/*λ* ratio of 0.90 (within the normal range). Blood and urine immunofixation electrophoresis demonstrated IgG *κ*–positive monoclonal protein.

The patient’s medical history included hypertension, coronary artery disease, and prior cerebral infarction. Physical examination revealed symmetrical mild pitting edema in both lower extremities.

Bone marrow evaluation showed 1% mature plasma cells on smear, and bone marrow biopsy revealed active hematopoietic proliferation with normal granulocyte-to-erythroid ratios. The granulocyte lineage consisted primarily of mid-to-late myeloid precursors, while erythroid lineage cells were present at all stages of development. The bone marrow biopsy confirmed normal plasma cell morphology and distribution.

### Renal biopsy

Renal biopsy yielded two small pieces of cortical tissue. The specimen contained 17 glomeruli, one of which exhibited global glomerulosclerosis.

Light microscopy: severe diffuse proliferation of mesangial cells and matrix was observed, presenting a nodular pattern (periodic acid-silver methenamine + Masson‘s trichrome stain). Substantial fuchsinophilic granular deposits were visible in the subendothelial space, with minimal deposition in the mesangial area. Approximately 25% of tubules demonstrated atrophy with corresponding interstitial fibrosis and lymphomononuclear cell infiltration. Three interlobular arteries were visible; no arcuate arteries were observed. Minimal haptoglobin deposition was noted subendothelially in the afferent arterioles. No crystal deposits were detected under polarized light microscopy. Congo red staining was negative.

Immunofluorescence on frozen tissue (IF-F): IgG (−), IgA (−), and IgM (+) in mesangial (MS) and segmental glomerular capillary wall (GCW), C1q (++) in MS and segmental GCW; C3 (+) in MS and segmental GCW; fibrin-related antigen (FRA) (−); *κ* (+); *λ* (−).

Immunofluorescence on paraffin-embedded tissue with antigen retrieval (IF-P): IgG1 (−), IgG2 (−), IgG3 (+ to ++), IgG4 (−), IgA (−), IgM (−), C1q (−), C3 (−), FRA (−), *κ* (+), and *λ* (−).

Electron microscopy: electron–dense deposits were identified in the subendothelial and mesangial regions without specific ultrastructural features. Colloidal gold immunoelectron microscopy demonstrated *κ* (+) and *λ* (−) reactivity.

**Figure 1:**
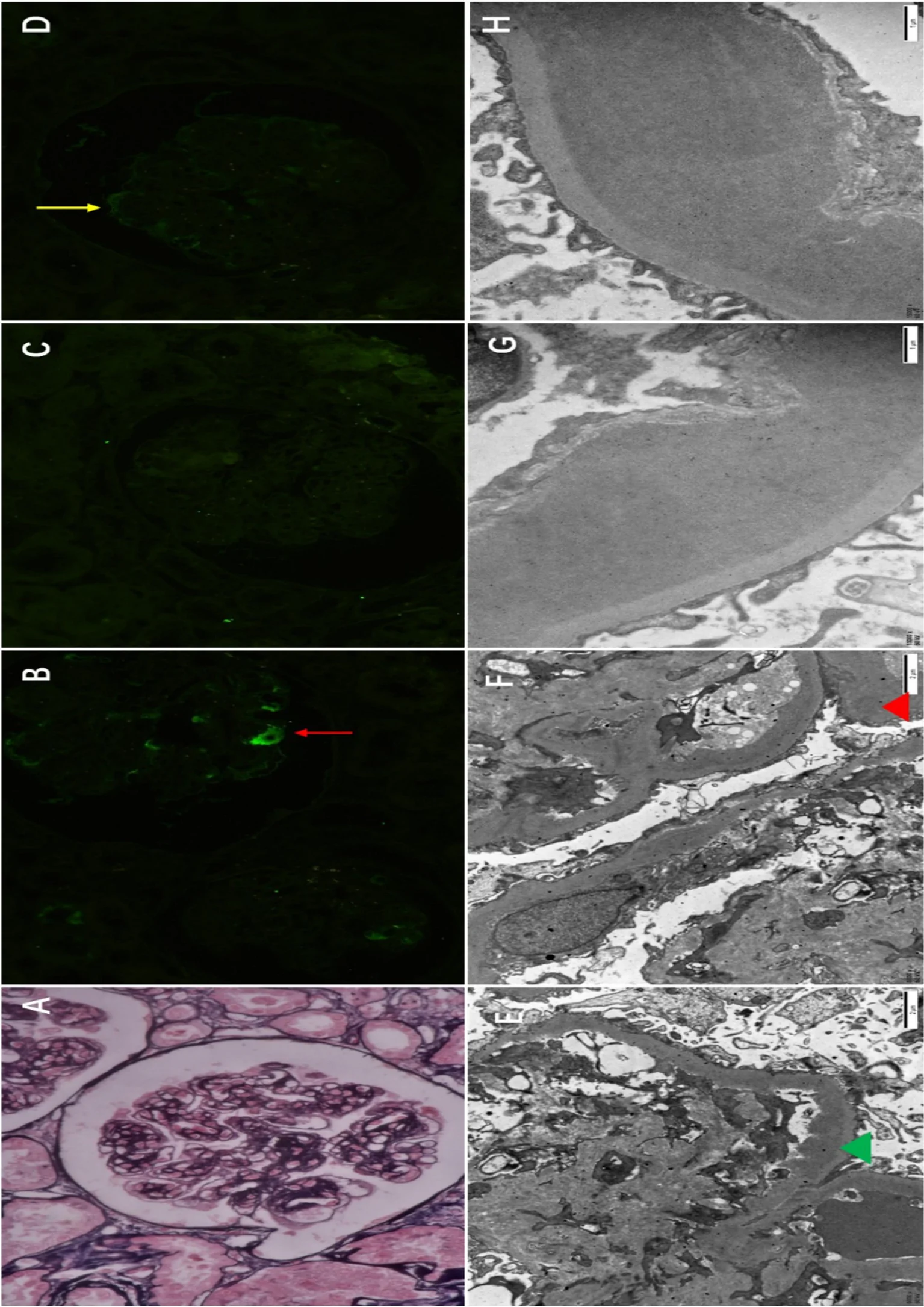
Renal biopsy findings of PGNMID with IgG3–*κ* deposits **(A–H)** images from the renal biopsy **(A)** Light microscopy reveals severe diffuse proliferation of mesangial cells and matrix, presenting a nodular pattern; substantial fuchsinophilic deposits are visible in the subendothelial space; minimal fuchsinophilic granular deposits are observed in the mesangial area, consistent with a membranoproliferative glomerulonephritis (MPGN) pattern of injury (periodic acid–silver methenamine + Masson’s Trichrome stain, 400×); **(B)** Paraffin immunofluorescence IgG3 (+ to ++); **(C)** Paraffin immunofluorescence *λ* (−); **(D)** Paraffin immunofluorescence κ (+); **(E)** There is moderate to severe proliferation of glomerular mesangial cells and mesangial matrix, accompanied by segmental homogeneous thickening of the glomerular basement membrane (GBM), and subendothelial electron-dense deposits; **(F)** Electron-dense deposits are observed in the mesangial region; **(G)** Colloidal gold immunoelectron microscopy κ (+); and **(H)** Colloidal gold immunoelectron microscopy λ (−).

These findings were consistent with proliferative glomerulonephritis with monoclonal immunoglobulin deposits (PGNMID IgG3–*κ*). The final diagnoses were PGNMID IgG3–*κ*, Stage 3a chronic kidney disease (CKD), Grade 3 hypertension, coronary artery disease, and prior cerebral infarction (Figure 1).

### Treatment and follow-up

According to the 2012 International Kidney Disease and Monoclonal Immunoglobulin Research Group (IKMG) treatment recommendations, chemotherapy was indicated given the patient’s serum creatinine level of 1.5 mg/dL (132.6 μmol/L), proteinuria of 5.61 g/24 h (classified as CKD Stage 3a), and age *>*65 years. Based on the patient’s weight (69 kg) and renal function (CKD Stage 3a), we administered four cycles of the CyBorD regimen: bortezomib 2.2 mg intravenously on days 1, 8, 15, and 22; cyclophosphamide 0.4 g intravenously weekly; and dexamethasone 20 mg intravenously on the days of bortezomib administration (age-reduced dose, 50% of the standard 40 mg dose). Concurrent antiviral prophylaxis with valacyclovir 500 mg daily was given to prevent herpes zoster reactivation. The patient had a 10-year history of hypertension and continued long-term valsartan therapy after the diagnosis of PGNMID.

**Figure 2:**
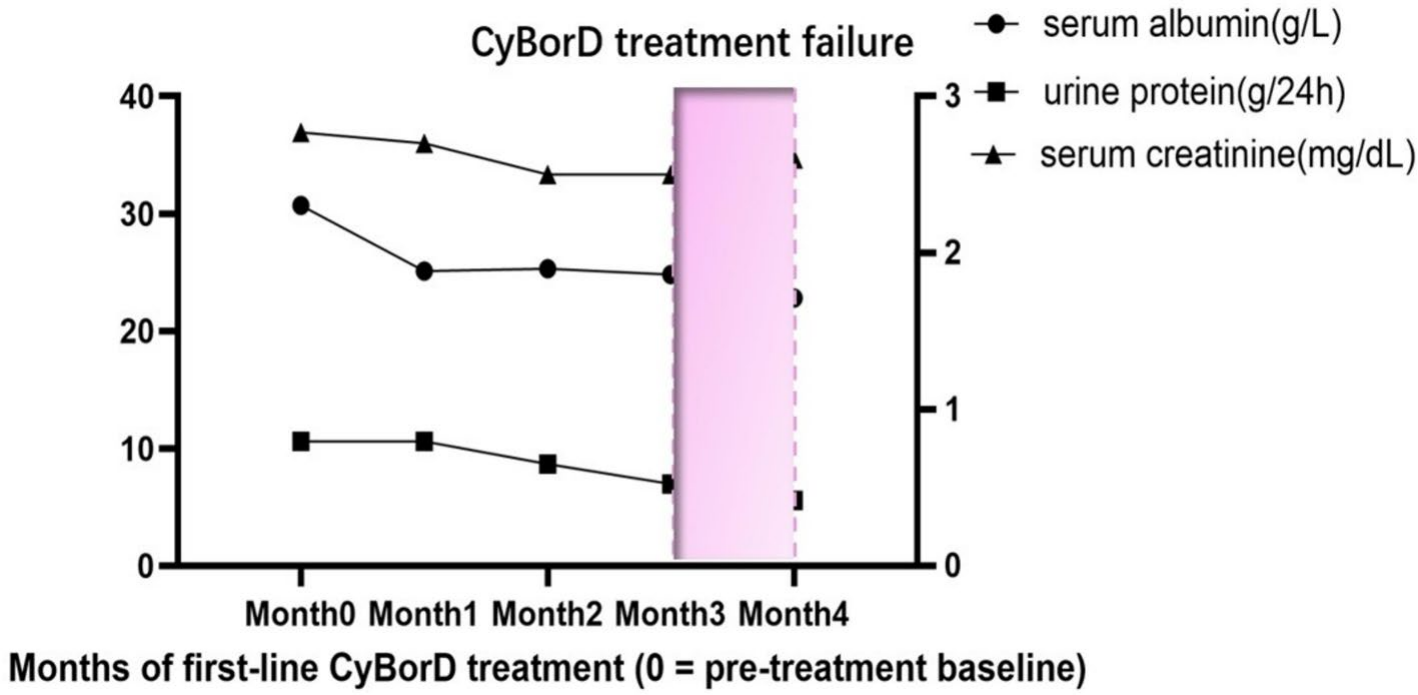
Clinical course during first–line CyBorD therapy. Longitudinal laboratory trends during the first-line CyBorD therapy (cyclophosphamide 0.4 g, bortezomib 2.2 mg, dexamethasone 20 mg). All laboratory units are indicated in the legend: Serum creatinine (mg/dL), 24-h urine protein (g/24 h), serum albumin (g/L). The *x*-axis shows cumulative treatment months. Progressive renal dysfunction, worsening nephrotic proteinuria and hypoalbuminemia emerged after four cycles, confirming CyBorD treatment failure.

Despite this treatment, the patient’s condition deteriorated. Serum creatinine levels ranged from 2.77 to 2.6 mg/dL (245 to 230 μmol/L), 24–hour urine protein ranged from 10.61 to 5.56 g, and serum albumin levels declined to 30.7–22.8 g/L. He also developed severe generalized edema and decompensated heart failure, the latter diagnosed based on clinical examination (dyspnoea, orthopnoea, pulmonary crackles, and peripheral oedema), chest X-ray (pulmonary congestion and pleural effusions), echocardiography, which revealed reduced left ventricular ejection fraction (LVEF) with global hypokinesis, and elevated NT-proBNP levels (>2,000 ng/L). Treatment failure was determined after completion of four cycles (approximately 16 weeks) based on progressive renal dysfunction, worsening proteinuria, declining serum albumin, and the development of decompensated heart failure. The patient was hospitalised and received intravenous furosemide (40–80 mg daily) for volume management. (Figure 2).

The standard dose of daratumumab is 16 mg/kg. Considering the patient’s advanced age and high infection risk, a risk-adapted regimen was implemented: Daratumumab 400 mg (∼6 mg/kg, 37.5% of the standard dose) was administered as a 4-h intravenous infusion on day 1 of each 30-day cycle, with subsequent infusions over 2–3 h if no infusion-related reactions occurred; bortezomib 2.2 mg (equivalent to 1.3 mg/m^2^) was given as an intravenous push on the same day. Premedication before each daratumumab infusion included dexamethasone 20 mg intravenously, calcium gluconate 1 g intravenously, and promethazine 25 mg intramuscularly. Infection prophylaxis consisted of valacyclovir 500 mg daily (herpes zoster) and trimethoprim-sulfamethoxazole 80/400 mg three times weekly (*Pneumocystis jirovecii* pneumonia). The patient completed all 12 planned cycles without dose reductions or delays. No infectious complications or other significant adverse events occurred during the 12-month treatment period. After 12 months of low-dose daratumumab-based therapy, clinical resolution of heart failure was documented, defined by disappearance of all symptoms and signs, normalisation of NT-proBNP (<400 ng/L), clearance of pulmonary congestion on follow-up chest X-ray, and improvement in LVEF on repeat echocardiography. The clinical deterioration—characterised by severe fluid overload, heart failure, and worsening hypoalbuminaemia—was primarily attributed to cumulative toxicities of the CyBorD regimen, particularly dexamethasone-induced fluid retention and cyclophosphamide-related toxicity, rather than to bortezomib resistance. This was supported by the absence of prior bortezomib exposure, the short duration of treatment (only four cycles), and the clinical pattern dominated by volume-related complications. Although the patient had failed CyBorD, we retained bortezomib in the salvage regimen because the failure was attributed to overall regimen toxicity rather than proven bortezomib resistance.

**Figure 3:**
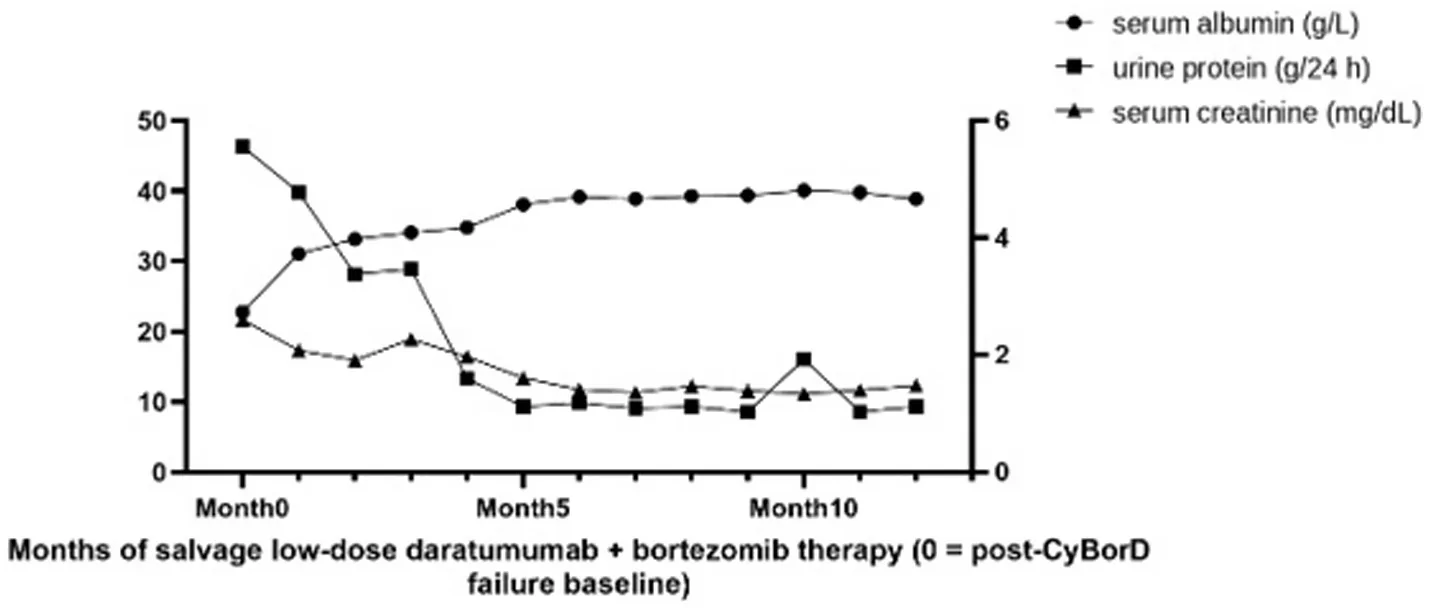
Serial laboratory responses during the low-dose daratumumab and bortezomib salvage treatment phase (daratumumab 400 mg ~ 6 mg/kg + bortezomib 2.2 mg monthly). All laboratory units are defined as follows: Serum creatinine (mg/dL), 24-h urine protein (g/24 h), serum albumin (g/L). The *x*-axis denotes months of salvage therapy starting from the baseline after CyBorD failure; the baseline data shows significant improvement in renal function, proteinuria and serum albumin compared with values at end of first-line CyBorD therapy.

After 12 months of treatment, substantial clinical improvement was observed: 24-h urine protein decreased to 1.12 g/24 h, serum albumin normalized to 38.1 g/L, and serum creatinine stabilized at 1.6 mg/dL (141.4 μmol/L). The patient achieved partial renal remission, and immunofixation electrophoresis converted to negative. Clinical resolution of heart failure was also documented. Resolution was confirmed the disappearance of all symptoms and signs, normalisation of NT-proBNP, and clearance of pulmonary congestion on follow-up chest X-ray (Table 1; Figure 3).

**Table 1:** Clinical and laboratory parameters before treatment, after CyBorD failure, and after 12 months of low-dose daratumumab-based therapy.

Parameter	Baseline (pre-treatment)	Post-CyBorD (treatment failure)	Post-daratumumab (12 months)
Serum creatinine (mg/dL)	1.5	2.7	1.6
24-h urine protein (g)	5.61	10.61	1.12
Serum albumin (g/L)	36.6	22.8	38.1
Immunofixation electrophoresis	IgG κ-positive	Positive	Negative
Clinical status	Stable	Severe oedema + heart failure	Clinical resolution

## Discussion and conclusions

Monoclonal immunoglobulin deposition-associated proliferative glomerulonephritis (PGNMID) is a rare kidney disease classified under the Monoclonal Immunoglobulin-related Kidney Syndrome (MGRS) category (2). It is characterized by localized renal involvement and chronic glomerular lesions, with a pathological presentation dominated by membranoproliferative glomerulonephritis. IgG3-*κ* is the most common immunophenotype in PGNMID, mainly due to the unique physicochemical properties of IgG3. IgG3 has a larger molecular weight, is less likely to pass through the glomerular filtration barrier, and exhibits strong Fc-Fc-mediated self-aggregation, leading to selective retention and deposition in the mesangium and subendothelial space. Moreover, IgG3 has the strongest complement-fixing ability, which triggers intense proliferative glomerular injury. In addition, *κ* light chains are more prevalent in monoclonal clones and structurally stable, facilitating typical granular deposits when combined with IgG3. These factors collectively make IgG3-κ the predominant phenotype in PGNMID (4), while electron microscopy reveals amorphous dense deposits. The clonal detection rate for this disease is only approximately 30%, and renal prognosis is poor, with 25% of patients progressing to end-stage renal disease (ESRD) within 30 months. Post-transplant recurrence rates are high; however, treatment centered on clonal-targeted chemotherapy can significantly improve renal outcomes (1, 4).

In this case, immunofluorescence on frozen tissue (IF-F) was entirely negative for immunoglobulins and light chains, which would have led to misdiagnosis as C3 glomerulopathy or unclassified MPGN (14). However, paraffin immunofluorescence (IF-P) with antigen retrieval successfully unmasked restricted IgG3-κ deposits, establishing the diagnosis of PGNMID. This discrepancy occurred because formalin fixation and paraffin embedding can cross-link or conceal immunoglobulin epitopes, and IF-F lacks an antigen retrieval step to restore their immunoreactivity (3, 14). In contrast, paraffin immunofluorescence (IF-P) with antigen retrieval (e.g., protease digestion) can effectively unmask epitopes masked by formalin fixation and protein cross-linking, greatly improving the detection of monoclonal IgG and light chains (15, 16). Recent studies confirm that IF-P is more sensitive than IF-F in MGRS-related renal lesions and masked monoclonal deposits, serving as an indispensable salvage and unmasking tool for IF-F–negative cases (3). This case highlights IF-P as an indispensable salvage technique when IF-F is negative but clinical suspicion for monoclonal gammopathy of renal significance remains high.

The IKMG (6) provided a therapeutic strategy for proliferative glomerulonephritis with monoclonal immunoglobulin deposits (PGNMID) that is centered on renal function staging and aims to eliminate the pathogenic B-cell or plasma cell clone. For patients with CKD stages 3–4, chemotherapy should be initiated, with a bortezomib-based regimen (cyclophosphamide + bortezomib + dexamethasone) as the first-line choice. The fundamental therapeutic principle of PGNMID is to eliminate pathogenic clonal plasma cells and reduce monoclonal immunoglobulin deposition to preserve renal function (7).

In a clone-directed case series of 19 patients with PGNMID, Gumber et al. demonstrated that targeted anti-plasma-cell therapy achieved an overall renal response rate of 88%, and no treated patient progressed to end-stage renal disease. In contrast, all untreated patients deteriorated to renal failure. These findings indicate that empirical anti-plasma cell therapy is highly beneficial even in patients without detectable clones, providing a critical rationale for managing serologically and hematologically occult cases (7). This patient was diagnosed with plasma cell clone–directed PGNMID (IgG3–κ), and was therefore treated with the CyBorD regimen. However, after four cycles of treatment, there was no improvement in the condition. The urine protein level increased to 10.61 g/24 h, serum albumin decreased to 22.8 g/L, creatinine rose to 2.7 mg/dL(238.6 μmol/L), and severe edema and heart failure were observed.

Daratumumab, as a humanized IgG1*κ* monoclonal antibody targeting CD38, exerts its core mechanism of action not only by inducing abnormal plasma cell apoptosis through antibody–dependent cellular cytotoxicity (ADCC), antibody–dependent cel- lular phagocytosis (ADCP), and complement–dependent cytotoxicity (CDC), but also by modulating the local renal immune microenvironment and eliminating immunosup- pressive cells, thereby creating favorable conditions for renal repair (9). Daratumumab is also recommended for the treatment of systemic light chain amyloidosis (10). Therefore, it holds promise as a potential treatment option for patients with PGNMID.

This drug is currently approved for the treatment of relapsed/refractory multiple myeloma, and its efficacy in the PGNMID setting is supported by clinical research. A recent open-label Phase II clinical trial showed that all 10 patients with PGNMID who received at least one infusion of daratumumab (16 mg/kg) achieved a renal response during the 12-month follow-up period, with 4 achieving complete remission (CR) and 6 achieving partial remission (PR). Five serious adverse events occurred among 162 infusions, including two cases of severe infectious complications. Interestingly, proteinuria and serum creatinine levels improved significantly after just a single infusion of daratumumab, with these improvements persisting for 12 months. Therefore, short-term or low-dose treatment may be a reasonable strategy for managing PGNMID (11). The Armani team conducted a small-scale study of PGNMID patients who had failed bortezomib therapy, enrolling a total of five female patients. After receiving daretocumab treatment, four of the five patients showed improvement, with three achieving renal remission (12). Xu et al. (17) reported on the potential of low-dose daratumumab combined with low-dose prednisone as a viable treatment option for patients with refractory PGNMID.

The selection of the 37.5% dose (400 mg, ~6 mg/kg) was primarily driven by the patient’s advanced age, heavy comorbidity burden (hypertension, coronary artery disease, prior cerebral infarction), and active decompensated heart failure, which rendered the standard 16 mg/kg regimen prohibitively risky for infusion-related reactions and infectious complications. Importantly, in PGNMID—a relatively low-tumour-burden monoclonal gammopathy of renal significance—full oncological dosing may not be strictly required. Indeed, Zand et al. (11) observed that a single standard-dose infusion could induce sustained renal responses for up to 12 months, supporting the pharmacological plausibility of dose de-escalation. The favourable outcome in our case, with partial renal remission and no infectious complications, provides preliminary support for this risk-adapted, low-dose strategy in this vulnerable population. Although daratumumab has been reported in PGNMID, including in patients with prior bortezomib-based therapy (11, 12), our case adds to the limited experience with a substantially reduced fixed monthly dose (400 mg, ~6 mg/kg, 37.5% of the standard dose) in a high-risk elderly patient with multiple comorbidities.

It is important to emphasise that the patient was not bortezomib-refractory; rather, the CyBorD regimen as a whole was poorly tolerated and ineffective in this patient due to a combination of disease progression and regimen-related toxicities. This distinction justifies the continuation of bortezomib in the salvage regimen, particularly given its synergistic anti-plasma-cell effects with daratumumab. A potential concern is the continued use of bortezomib despite prior CyBorD failure. We acknowledge that this requires clarification. The failure was regimen-specific rather than bortezomib-specific. The patient’s deterioration was characterized by fluid overload, heart failure, and hypoalbuminemia—complications that limited the delivered dose intensity of all three agents. Cyclophosphamide-related toxicity (myelosuppression, hemorrhagic cystitis) and dexamethasone-induced fluid retention likely contributed more to clinical decline than bortezomib ineffectiveness. Moreover, daratumumab and bortezomib have synergistic anti-plasma cell effects through complementary mechanisms: daratumumab mediates ADCC, ADCP, and CDC, while bortezomib induces proteasome inhibition and alters the tumor secretory profile. By removing cyclophosphamide and reducing dexamethasone, the salvage regimen offers a distinctly different toxicity profile (9).

It is important to recognise that the patient received daratumumab in combination with bortezomib, and that cyclophosphamide and high-dose dexamethasone were withdrawn concurrently. Optimisation of diuretic therapy, control of congestion, and continuation of valsartan may also have contributed to the observed improvement. Therefore, the response cannot be attributed solely to low-dose daratumumab. In this single case, the respective contributions of each intervention cannot be disentangled, and the observed improvement should be interpreted as a temporal association with the overall change in management rather than as a proven effect of daratumumab alone. In this case, the treatment regimen of low-dose daratumumab combined with bortezomib achieved favorable clinical outcomes. After receiving four doses of daratumumab, the patient’s blood immunofixation electrophoresis results turned negative, urinary protein levels decreased significantly, serum albumin levels rose markedly, serum creatinine returned to baseline levels, and the patient’s edema and heart failure were resolved; After 12 months of treatment, 24-h urine protein quantification decreased to 1.12 g/24 h, serum albumin rose to 38.1 g/L, and serum creatinine decreased to 1.6 mg/dL (141.4 μmol/L), indicating partial remission of nephropathy. Furthermore, no adverse reactions, such as infections, occurred during the treatment period, supporting the potential feasibility of this low-dose regimen in this patient. Furthermore, this case demonstrates that renal damage in PGNMID patients is partially reversible following effective targeted therapy. Even in stage 3a CKD with impaired renal function, timely inhibition of monoclonal immunoglobulin deposition can still reduce glomerular inflammation and proliferative damage, thereby preserving residual renal function. This provides clinical evidence for the importance of early intervention and optimal timing of treatment in PGNMID. This case illustrates that a low-dose daratumumab-containing salvage regimen, combined with bortezomib and optimised supportive care, was temporally associated with partial renal remission in a high-risk elderly patient with CyBorD-refractory PGNMID. However, the specific contribution of daratumumab cannot be isolated from the concurrent interventions, and our findings should be considered hypothesis-generating rather than definitive.

Several limitations of this case report should be acknowledged: (1) CD38 + cell percentage and B-cell subpopulation analysis were not performed during treatment, which limited further exploration of the mechanistic rationale for low-dose daratumumab efficacy; (2) renal rebiopsy was not performed at the end of treatment due to the patient’s fragile cardiac function, which made it impossible to evaluate the pathological improvement of glomerular deposits; (3) this is a single case report with a relatively short follow-up period (12 months), and the long-term efficacy and relapse rate of the low-dose regimen need to be confirmed by longer follow-up and more cases; (4) the lack of a control group makes it impossible to directly compare the efficacy and safety of low-dose and standard-dose daratumumab in PGNMID-IgG3*κ* patients. Despite these limitations, this case provides valuable clinical evidence for the use of low-dose daratumumab combined with bortezomib in elderly, high-risk patients with PGNMID-IgG3κ, —a disorder traditionally classified within the MGRS spectrum, though recent evidence has raised the possibility that some cases may represent an immune-mediated oligoclonal glomerulopathy rather than a conventional MGRS entity (13); (5) the daratumumab-containing regimen was combined with other concurrent interventions, making the specific contribution of each component impossible to isolate.

In summary, this case demonstrates that low-dose daratumumab (400 mg, ∼6 mg/kg, 37.5% of the standard dose) combined with bortezomib is a potentially feasible and apparently well-tolerated salvage option for elderly, high-risk patients with CyBorD-refractory PGNMID and multiple comorbidities. The patient achieved partial renal remission with clinical resolution of heart failure and no infectious complications over 12 months of treatment. This favorable outcome suggests that dose deescalation of daratumumab may preserve therapeutic efficacy while mitigating toxicity in vulnerable populations. Although further prospective studies are needed to define the minimal effective dose and optimal treatment duration, our experience suggests that reduced-dose daratumumab may be worth investigating further in elderly patients with refractory PGNMID who are poor candidates for standard-dose regimens.

## Data Availability

The original contributions presented in the study are included in the article/supplementary material, further inquiries can be directed to the corresponding author.

## Glossary

ADCC: antibody–dependent cellular cytotoxicity
ADCP: antibody–dependent cellular phagocytosis
CDC: complement–dependent cytotox-icity
CKD: chronic kidney disease
CR: complete remission
CyBorD: cyclophos- phamide, bortezomib, and dexamethasone
ESRD: end–stage renal disease
FRA: fibrin–related antigen
GCW: glomerular capillary wall
IgA: immunoglobulin A
IgG: immunoglobulin G
IgG1: immunoglobulin G1
IgG2: immunoglobulin G2
IgG3: immunoglobulin G3
IgG4: immunoglobulin G4
IgM: immunoglobulin M
IKMG: international kidney and monoclonal gammopathy research group
MGRS: mono-clonal gammopathy of renal significance
MM: multiple myeloma
MN: membranous nephropathy
MPGN: membranoproliferative glomerulonephritis
MS: mesangial
MsPGN: mesangial proliferative glomerulonephritis
NR: no response
PGNMID: proliferative glomerulonephritis with monoclonal immunoglobulin deposits
PR: partial remission
*κ*: kappa light chain
*λ*: lambda light chain
LVEF: left ventricular ejection fraction
GBM: glomerular basement membrane
